# Effect of two vitamin D repletion protocols on 24-h urine calcium in patients with recurrent calcium kidney stones and vitamin D deficiency: a randomized clinical trial

**DOI:** 10.1186/s40001-023-01226-z

**Published:** 2023-07-22

**Authors:** Lilit Sardari Masihi, Nasrin Borumandnia, Maryam Taheri, Abbas Basiri, Hossein Imani, Saba Jalali, Sanaz Tavasoli

**Affiliations:** 1 Department of Clinical Nutrition, School of Nutritional Sciences and Dietetics, Tehran University of Medical Sciences (TUMS), Tehran, Islamic Republic of Iran; 2 Urology and Nephrology Research Center, Shahid Beheshti University of Medical Sciences, No.103, Shahid Jafari St., Pasdaran Ave., Tehran, 1666668111 Islamic Republic of Iran; 3 Human Nutrition, Faculty of Land and Food Systems, University of British Columbia, Vancouver, CA USA

**Keywords:** Vitamin D deficiency, Urolithiasis, 24-h urine, Hypercalciuria

## Abstract

**Objectives:**

To evaluate the effects of two vitamin D repletion therapies (cholecalciferol) on serum levels of 25-hydroxyvitamin D (25(OH)D) and 24-h urine calcium in patients with recurrent calcium kidney stones and vitamin D deficiency (VDD).

**Design, setting, participants:**

A parallel-group randomized controlled clinical trial on patients who referred to Labbafinejad kidney stone prevention clinic, Tehran, Iran. From 88 recurrent calcium stone formers, 62 patients completed the study. The age of participants was 18–70 years who had serum 25(OH)D levels of 10–20 ng/ml.

**Intervention:**

Participants received oral cholecalciferol 2000 IU daily for 12 weeks or 50,000 IU weekly for 8 weeks.

**Main outcome measures:**

Study variables including 24-h urine calcium, supersaturations of calcium oxalate and calcium phosphate, serum 25(OH)D and parathyroid hormone were measured at the beginning of the study and after 12 weeks.

**Results:**

The 24-h urine calcium significantly increased in both groups (β = 69.70, p < 0.001), with no significant difference between treatments. Both groups showed no significant change in the supersaturation levels of calcium oxalate and calcium phosphate. Serum levels of 25(OH)D increased significantly (β = 12.53, p < 0.001), with more increase in the 50,000 IU group (β = 3.46, p = 0.003). Serum parathyroid hormone decreased in both groups (p < 0.001).

**Conclusions:**

Although both treatment protocols increased 24-h urine calcium, they did not increase the supersaturation state of calcium oxalate or calcium phosphate.

*Trial registration* IRCT20160206026406N4, 13/08/2019.

## Introduction

Prevalence of vitamin D deficiency (VDD) in kidney stone formers is 18.9 to 59% [1]. In a case–control study by Ticinesi et al. (884 patients with idiopathic calcium stones vs. 967 non-stone-forming controls), the prevalence of VDD (< 20 ng/ml) was 56% in stone formers and 44% in control group (p < 0.001) [2]. In another case–control study on 239 calcium stone formers vs. 127 non-stone-forming controls [3], a VDD was observed in 28% of the patients versus 15.7% in controls (p = 0.009).

However, due to conflicting results of studies regarding association between serum 25(OH)D and hypercalciuria, there has been a concern for VDD treatment in calcium stone formers [4]. Furthermore, few studies evaluated the effect of VDD treatment on hypercalciuria in kidney stone formers [5–10]. These studies used different treatment protocols and found conflicting results; therefore, there is a lack of sufficient and robust evidence about the safety of VDD treatment in patients with kidney stones [11]. In the current study, we aimed to investigate the effects of two treatment protocols on serum levels of 25(OH)D and 24-h urine calcium (24-U Ca) in patients with recurrent calcium kidney stones and VDD.

## Methods

### Study design and participants

This study was a parallel-group randomized controlled clinical trial undertaken in the kidney stone prevention clinic of Shahid Labbafinejad medical center, from April 2018 to May 2020. The patients were recurrent calcium stone formers who had serum 25-hydroxyvitamin D (25(OH)D) level of 10–20 ng/mL, aged 18–70 years, had body mass index (BMI) of less than 30 kg/m^2^ [12], 24-U Ca below 300 mg [6], normal serum calcium levels who agreed to participate in the study. The recurrent calcium kidney stone was defined as having at least two episodes of radiopaque stones in the past medical history of the patient [13, 14]. The following cases were not included in our study: patients who were pregnant or lactating, using calcium supplements or other forms of vitamin D supplements or any medical drug which may affect serum or urinary calcium, had any history of kidney stone passage or gross hematuria within two months before the study, had known history of primary hyperparathyroidism or diseases that affect vitamin D and calcium metabolism (such as sarcoidosis or some other chronic granulomatous disorders), diabetes mellitus, and any malignancy or malabsorption. Patients who had 24-h urine volume under or over-collection [15] were also excluded from the study.

The study was approved by the ethics committee of the Urology and Nephrology Research Center, Shahid Beheshti University of Medical Sciences (IR. SBMU.UNRC.1395.26), and Tehran University of Medical Sciences (IR.TUMS.VCR.REC.1397.193). The trial was registered in the Iranian Registry of Clinical Trials (IRCT) (IRCT20160206026406N4). All the study protocols were performed in accordance with the 1964 Declaration of Helsinki, and all participants gave written informed consent before the study.

### Study interventions and outcomes

Study participants received either a daily dose of 2000 IU oral cholecalciferol for 12 weeks (maintenance dose) or a weekly dose of 50,000 IU oral cholecalciferol for eight weeks (loading dose). The final assessment was performed 12 weeks after the beginning of the study. These doses were selected according to loading treatment and maintenance therapy recommendations for VDD management, published by the endocrine society clinical practice guidelines [16]. In addition, all patients received general nutritional consultation for preventing kidney stone recurrence according to the European Association of Urology guidelines [17] with emphasis on taking adequate calcium from dairy products (800–1200 mg/day), salt intake restriction to less than 5 g/day, and consumption of moderate amounts of animal proteins (0.8–1 g/kg/day) [18–20].

Because of the differences in the study protocol, participants and researchers were not blind to the study protocol, and only the statistician was blinded for the statistical analyses.

Study variables included demographic, anthropometric, the duration and episodes of kidney stone disease, serum 25(OH)D, parathyroid hormone (PTH), calcium, and phosphate concentrations, 24-h urine analysis for volume and concentration of urea, creatinine, calcium, sodium, potassium, phosphate, magnesium, citrate, oxalate and uric acid, and also participant`s dietary intake data. Demographic, anthropometric, and kidney stone history data were collected at the beginning of the study. Serum and 24-h urine samples were taken at the beginning and the end of study. All the laboratory measurements were performed as we reported in our previous study [9]. The LithoRisk® software (Biohealth, Italy) was used to calculate relative supersaturations of calcium salts, i.e., calcium oxalate (CaOxSS) and calcium phosphate (CaPSS). Dietary intakes were assessed by two 24-h dietary recalls on non-consecutive days at the beginning, the sixth week of intervention, and the end of study. The recalls were analyzed using Nutritionist 4 software (N-Squared Computing), modified according to Iranian food composition.

### Statistical methods

The sample size was calculated using the following equation:

$${\text{n}} \ge \frac{{2(Z_{1 - \alpha /2} + Z_{1 - \beta } )^2 \sigma^2 }}{{\left( {\mu_0 - \mu_1 } \right)^2 }}$$

Considering the type one error of 0.05, type 2 error of 0.20, and $$\begin{array}{*{20}c} {effect\begin{array}{*{20}c} {\,} \\ \end{array} \begin{array}{*{20}c} {size = \frac{\mu_1 - \mu_2 }{\sigma }} \\ \end{array} } \\ \end{array}$$ of 0.75, the minimum required sample size was 28 in each group. Patients were randomized into study groups with an allocation ratio of 1:1 with a simple randomization method. A random sequence was generated using a computer program to produce the comparable groups and eliminate the source of bias in treatment assignments.

All the statistical analyses were performed using SPSS software version 24.0 (IBM, Chicago, Illinois, USA). Data are shown as mean ± standard deviation or median (IQR) for quantitative variables and frequency (percentage) for qualitative ones. The Kolmogorov–Smirnov test was used to assess the normality of data distribution. According to normality test results, the independent sample T-test or Mann–Whitney test was used to compare the mean outcome quantities between the two study groups. The Chi-Square test or Fisher exact test were also used to compare qualitative factors between the two groups. Serum variables, 24-h urine analysis, and supersaturation changes were compared between groups using a univariate general linear model (GLM) with generalized estimating equations (GEE) approach. Finally, multivariable GEE analysis was performed to assess the 24-h urinary calcium changes by adjusting confounding variables. Regression coefficient (β) were reported along with 95% confidence interval. The β (regression coefficient) signifies how much the mean of a dependent variable (for example the level of serum parathyroid hormone) changes when the participants received loading vitamin D repletion therapy (50,000 IU cholecalciferol/oral/weekly) compared with the maintenance vitamin D repletion protocol (2000 IU cholecalciferol/oral/daily). A p- value < 0.05 was considered statistically significant.

## Results

**Fig. 1:**
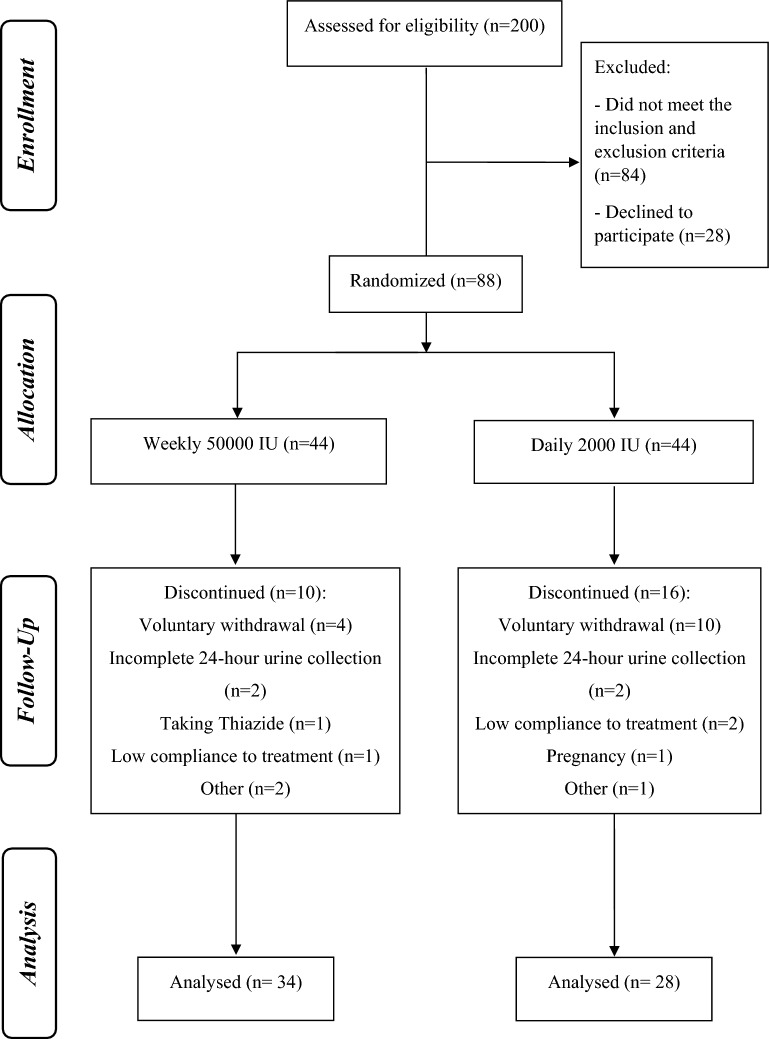
Flow diagram for participants included in the study

Eighty-eight patients were randomized to study groups, with participation rate of 44%. Of them, 34 patients in the 50,000 IU group and 28 patients in the 2000 IU group completed the study (Fig. 1).

Baseline characteristics for 62 participants who completed the study are shown in Table 1. Duration of kidney stone history was significantly different between patients (p = 0.027). Other parameters had similar distribution between two groups (p > 0.05). The average amount of dietary intake showed no significant difference between groups in terms of energy, macronutrients and micronutrients including vitamin D and calcium (p > 0.05).

**Table 1:** Baseline characteristics of participants

	Study subgroups		P- value
	2000 IU/daily	50,000 IU/weekly	
Age, years, mean (SD)	45.75 (10.88)	47.03 (11.20)	0.652^†^
*Gender, Number (percentage)*			
Female	9 (32.1)	6 (17.6)	0.398^‡^
Male	19 (67.9)	28 (82.4)	
*Family history of urolithiasis, Number (percentage)*			
Yes	19 (67.9)	19 (55.9)	0.340^‡^
No	9 (32.1)	15 (44.1)	
Kidney stone duration, years, median (IQR)	14.50 (4.00, 25.00)	5.00 (1.50, 12.00)	0.027^*§^
Stone episode, number, median (IQR)	4.00 (2.50, 7.50)	2.00 (1.00, 5.00)	0.170^§^
BMI, Kg/m2, mean (SD)	27.55 (2.66)	27.10 (2.33)	0.476^†^

Results are expressed according to variable distribution. ^†^Independent T test, ^‡^Chi square Test

^§^Mann–Whitney test. ^*^ p < 0.05. Bold values emphasize statistical significance. Abbreviation: IQR, Inter Quartile Range; BMI, Body Mass Index; SD, Standard Deviation

Table 2 represents the serum variables of both groups at the start and the end of the study. Both treatment protocols significantly increased serum 25(OH)D levels (p < 0.001). Comparing the changes in groups, increase in 50,000 IU group was more than 2000 IU group (β: 3.46, 95% CI 1.21, 5.70, p = 0.003). Considering normalization of serum vitamin D, serum 25(OH)D reached normal level (≥ 30 ng/mL [16]) in 55.9% of participants in 50,000 IU group and 25% of participants in 2000 IU group. Regarding other serum variables, PTH decreased (p < 0.001), and phosphate increased (p = 0.025) in both groups, with no significant difference between the two treatment protocols (p = 0.675, p = 0.345, respectively). In addition, we assessed the correlation between 24-U Ca alterations with serum 25(OH)D and PTH changes. There was no significant correlation between 24-U Ca and serum 25(OH)D level differences (r = 0.28, p = 0.15; r = 0.10, p = 0.56 in 2000 IU/daily and 50,000 IU/weekly, respectively). It is interesting that the correlation was higher in groups which received 2000 IU vitamin D per day. In addition, the correlation between 24-U Ca and serum PTH differences was not significant in both groups (r = − 0.14, p = 0.48; r = − 0.13, p = 0.47 in 2000 IU/daily and 50,000 IU/weekly, respectively).

**Table 2:** Serum variables of the study groups at the baseline and the end of intervention. The groups were compared using general linear model (GLM) with generalized estimating equations (GEE) approach

	Study groups				Time effect		Group effect	
	2000 IU/daily		50,000 IU/weekly		β^†^ (95% CI)	p- value	β (95% CI)	p- value
	Before	After	Before	After				
25(OH)D (ng/mL)	16.24 (3.05)	24.82 (7.75)	16.09 (3.50)	31.88 (8.85)	12.53 (10.26, 14.80)	< 0.001^***^	3.46 (1.21, 5.70)	0.003^**^
PTH (pg/mL)	56.23 (16.93)	48.84 (16.15)	54.35 (19.22)	47.37 (15.80)	− 7.16 (− 10.23, − 4.08)	< 0.001^***^	3.98 (− 9.48, 6.13)	0.675
Calcium (mg/dl)	9.31 (0.32)	9.51 (0.48)	9.34 (0.45)	9.38 (0.42)	0.11 (− 0.02, 0.25)	0.112	− 0.05 (-.20, 0.10)	0.516
Phosphate (mg/dl)	3.25 (0.52)	3.47 (0.74)	3.35 (0.54)	3.56 (0.49)	0.21 (0.02, 0.41)	0.025^*^	0.10 (− 0.10, 0.30)	0.345
Creatinine (mg/dl)	1.12 (0.24)	1.07 (0.16)	1.10 (0.20)	1.12 (0.18)	− 0.009 (− 0.05, 0.03)	0.690	0.01 (− 0.07, 0.10)	0.743

Before and after values in the table are shown as mean (standard deviation)

CI, confidence interval; 25(OH)D, 25-hydroxyvitamin D; PTH, parathyroid hormone; BUN, Blood urea nitrogen

^*^p < 0.05; **p < 0.01; ***p < 0.001. Bold values emphasize statistical significance

^†^Regression coefficient (β) were reported along with 95% confidence interval. The β (regression coefficient) signifies how much the mean of a dependent variable (for example the level of serum parathyroid hormone) changes when the participants received loading vitamin D repletion therapy (50,000 IU cholecalciferol/oral/ weekly) compared with the maintenance vitamin D repletion protocol (2000 IU cholecalciferol/oral/daily)

The results of 24-U analysis and calculated supersaturations of participants are shown in Table 3. The 24-U Ca significantly increased in both groups during the time (β = 69.70, 95% CI 52.34, 87.06, p < 0.001), but we found no significant difference between two groups (p = 0.602). Three patients in the 2000 IU group (10.7%) and six patients in the 50,000 IU group (17.6%) had 24-U Ca more than 300 at the end of the study, which was not significantly different (p = 0.494). None of the study groups showed a significant change in CaOx SS and CaP SS (p > 0.05).

**Table 3:** The results of 24-h urine analysis and supersaturation indices of the study groups at baseline and the end of the treatment by vitamin D supplement (cholecalciferol)

	Study groups				Time effect		Group effect	
	2000/daily		50,000/weekly		β (95% CI)	p- value	β (95% CI)	p- value
	Before	After	Before	After				
24-U volume (mL/24 h)	2098 (898)	2414 (1094)	1806 (687)	2196 (768)	357 (174, 539)	< 0.001^***^	− 255 (− 653, 144)	0.210
24-U creatinine (gr/24 h)	1.18 (0.35)	1.19 (0.35)	1.16 (0.37)	1.22 (0.31)	0.03 (− 0.03, 0.10)	0.296	0.003 (− 0.15, 0.16)	0.966
24-U calcium (mg/24 h)	160.29 (62.07)	233.50 (64.90)	155.26 (67.81)	222.06 (93.56)	69.70 (52.34, 87.06)	< 0.001^***^	− 8.23 (− 39.18, 22.72)	0.602
24-U sodium (meq/24 h)	134.48 (74.83)	154.90 (53.65)	159.38 (64.41)	156.29 (57.29)	7.52 (− 11.32, 26.38)	0.434	13.14 (− 11.21, 37.50)	0.290
24-U potassium (meq/24 h)	49.98 (16.71)	53.07 (17.45)	49.86 (24.77)	57.08 (27.16)	5.35 (− 0.98, 11.68)	0.098	1.96 (− 6.72, 10.61)	0.660
24-U phosphate (gr/24 h)	0.66 (0.24)	0.73 (0.26)	0.65 (0.23)	0.71 (0.28)	0.06 (− 0.008, 0.13)	0.085	− 0.01 (− 0.11, 0.08)	0.750
24-U magnesium (mg/24 h)	64.47 (21.46)	81.31 (34.14)	65.41 (24.85)	92.05(35.58)	22.21 (14.03, 30.39)	< 0.001^***^	5.83 (− 6.24, 17.92)	0.344
24-U citrate (mg/24 h)	741.26 (352.89)	810.64 (348.32)	626.44 (337.74)	891.38 (373.75)	181.27 (99.33, 263.20)	< 0.001^***^	− 11.89 (− 167.71, 143.92)	0.881
24-U oxalate (mg/24 h)	41.11 (15.71)	43.90 (18.76)	37.09 (15.98)	43.00 (18.49)	4.50 (0.14, 9.14)	0.058	− 2.46 (− 9.53, 4.60)	0.494
24-U urea (gr/24 h)	27.07 (9.04)	30.63 (8.62)	27.94 (9.71)	30.59 (9.56)	3.06 (0.44, 5.67)	0.022*	0.41 (− 3.26, 4.08)	0.827
24-U uric acid (mg/24 h)	431.54 (146.79)	416.78 (139.60)	467.09 (216.64)	478.56 (175.28)	− 0.37 (− 46.56, 45.56)	0.987	48.66 (− 22.44, 119.77)	0.180
pH	5.39 (0.83)	5.50 (0.96)	5.12 (0.48)	5.38 (0.82)	0.19 (− 0.05, 0.43)	0.120	− 0.19 (− 0.50, 0.10)	0.207
CaOx SS	5.52 (2.83)	6.23 (2.13)	5.27 (2.42)	6.05 (2.60)	0.74 (− 0.00, 1.50)	0.052	− 0.22 (− 1.19, 0.75)	0.654
CaP SS	0.58 (1.16)	0.58 (1.19)	0.20 (0.24)	0.68 (1.45)	0.26 (− 0.06, 0.58)	0.110	− 0.13 (− 0.59, 0.31)	0.550

The groups were compared using general linear model (GLM) with generalized estimating equations (GEE) approach. The values of before and after of study groups stand for mean (standard deviation)

CI, confidence interval; 24-U, 24-Hour urine; CaOx SS, calcium oxalate supersaturation; CaP SS, calcium phosphate supersaturation

^*^p < 0.05; **p < 0.01; ***p < 0.001. Bold values emphasize statistical significance

In addition to univariate analyses, a multivariate GEE model was used to adjust the effect of potential confounders in evaluation of the treatment protocols on urinary calcium (Table 4). Similar to univariate results, after adjusting confounding variables, the amount of 24-U Ca increased during the time in both groups (β = 44.01 95% CI 16.49, 71.54, p = 0.002), with no significant difference between two groups (p = 0.664).

**Table 4:** Evaluation of 24-h urinary calcium changes by modulating the effects of confounding variables

Variables	β^†^ (95% CI)	p- value
*Study group*		
50,000 IU/weekly	-7.485 (− 41.27, 26.30)	0.664
2000 IU/daily	Reference	
*Time*		
After treatment	44.014 (16.48, 71.54)	0.002^**^
Before treatment	Reference	
*Gender*		
Male	− 14.955 (− 52.40, 22.49)	0.434
Female	Reference	
Age, years	-1.436 (− 2.93, 0.06)	0.061
24-U citrate (mg/24 h)	0.025 (− 0.01, 0.06)	0.175
24-U sodium (mg/24 h)	0.212 (− 0.01, 0.43)	0.062
Serum PTH (pg/mL)	− 0.199 (− 0.91, 0.51)	0.586
Serum 25(OH)D (ng/mL)	1.424 (− 0.66, 3.51)	0.182
Dietary calcium (mg)	− 0.001 (− 0.05, 0.05)	0.977
Dietary protein intake (gr)	− 0.259 (− 1.38, 0.87)	0.653
Protein food group	− 0.259 (− 1.38, 0.87)	0.653
Kidney stone duration, years	0.909 (− 0.79, 2.61)	0.297

CI, confidence interval; PTH, parathyroid hormone; 25(OH)D, 25-hydroxyvitamin D

^**^p < 0.01. Bold values emphasize statistical significance

^†^Regression coefficient (β) were reported along with 95% confidence interval. The β (regression coefficient) signifies how much the mean of a dependent variable (for example the level of serum parathyroid hormone) changes when the participants received loading vitamin D repletion therapy (50,000 IU cholecalciferol/oral/ weekly) compared with the maintenance vitamin D repletion protocol (2000 IU cholecalciferol/oral/daily)

## Discussion

Although some guidelines, such as the Canadian Urology Association, recommend repletion therapy for kidney stone forming patients with VDD [21], there are still concerns about the best treatment protocol in these patients [7]. With this aim in view, our study evaluated the effects of two treatment protocols, i.e., loading and maintenance vitamin D repletion therapy, on 24-U Ca. The loading protocol in the current study was weekly dose of 50,000 IU oral cholecalciferol for 8 weeks, which is a standard therapy protocol for VDD. The maintenance protocol was daily dose of oral cholecalciferol 2000 IU for 12 weeks, recommended for people at higher risk for VDD. Since the groups did not have equivalent cumulative dosage (400,000 IU vs. 168000 IU), we did not expect similar serum 25(OH)D increase in study groups. Our results showed that serum 25(OH)D levels increased in both groups, with a higher increase in 50,000 IU group.

Vitamin D is crucial to improve the efficiency of calcium absorption in the gut. When VDD exists, calcium absorption declines. Consequently, the level of serum PTH increases (called secondary hyperparathyroidism) to maintain serum calcium through increasing renal tubular calcium reabsorption and bone resorption [16, 22]. The treatment of VDD leads to normalization of serum 25(OH)D and PTH. Consequently, an increase in intestinal calcium absorption and decrease in renal tubular calcium reabsorption occur. Therefore, an increase in urinary calcium is speculated after VDD treatment and PTH normalization [7]. The main concern about vitamin D repletion is the change in urinary calcium as a promoter of kidney stone formation.

The results of studies evaluating the effects of vitamin D supplementation on urinary calcium in urolithiasis patients are controversial (Table 5) [5–10]. Most of these studies had limitations, including non-controlled design of trials [6–9], retrospective design [9, 10], and small sample size [5]. Therefore, randomized controlled clinical trials with higher sample sizes are needed to clarify this conflict.

**Table 5:** Some Studies on the effect of vitamin D repletion therapy on the 24-hour urine calcium in kidney stone formers

References	Study methodology	Vitamin D repletion dose	Sample size	Baseline 25-OH D (ng/ml)	Study time	24-Urine calcium (mg/day)	Other main findings 25-OH D (ng/ml) PTH
Vitale et al. [7]	Retrospective	Oral bolus of 100,000–200,000 IU; followed by 5000–10,000 IU/weekly OR, 25,000–50,000 IU/monthly	33	< 20	6 months	Significant increase (p < 0.01)	Significant 25(OH)D mean increase; 11.8 to 40.2 (p < 0.01) Significant PTH decrease (p < 0.01)
Ganji et al. [8]	Non-controlled clinical trial	50,000 IU/weekly/ for 8 weeks followed by 50,000 IU/ every forth night	30	< 30	3 months	No significant increase (p = 0.39)	Significant 25(OH)D mean increase; 10.4 to 44.0 (p < 0.001) Significant PTH decrease (p < 0.001)
Taheri et al. [9]	Retrospective	50,000 IU/weekly	26	< 30	8–12 weeks	Significant increase (p < 0.001)	Significant 25(OH)D mean increase from 14.1 to 33.6 (p < 0.001) Significant PTH decrease (p < 0.001)
Ferroni et al. [5]	Randomized controlled trial	1,000 IU/daily (8 patients) OR, 50,000 IU/weekly (13 patients)	21	< 30	6 weeks	No significant increase^§^ (p > 0.05)	Significant 25(OH)D increase only in 50,000 IU group (p < 0.01) PTH: Not evaluated
Hesswani et al. [10]	Retrospective	1000 IU Vitamin D/daily + 945 mg Calcium /daily^†^	34	< 30	39 months^‡^	Significant increase (p < 0.001)	Significant 25(OH)D mean increase from 20.83 to 26.60, (p < 0.001) No significant PTH change (p = 0.98)
Leaf et al. [6]	Non-controlled clinical trial	50,000 IU/weekly	29	< 30	8 weeks	No significant increase (p = 0.91)	Significant 25(OH)D mean increase from 17 to 35, (p < 0.001) No significant PTH change (p = 0.71)

All of these studies didn’t have any dietary assessment

25(OH)D, 25-hydroxyvitamin D; PTH, parathyroid hormone

^†^The range of vitamin D and calcium were 400–1500 IU/daily and 315–1500 mg/daily, respectively

^‡^The range of follow-up time was 7 to 60 months

^§^24-urine calcium changed from 231 mg/day to 219 mg/day and from 195 mg/day to 263 mg/day in group with vitamin D dose of 1,000 IU/ per day or 50,000 IU/per week, respectively

Our study results showed that both treatment protocols increased 24-U Ca by about 44 mg/24 h, with no difference between the two groups. Comparing with previous studies (Table 5), our finding is consistent with the results of Vitale et al. [7], Taheri et al. [9], and Hesswani et al. [10]. Conversely, it is against the result of Ganji et al. [8], Ferroni et al. [5], and Leaf et al. [6]. The reasons mentioned in these studies as the cause for increased urinary calcium include simultaneous calcium supplementation [10], the effect of confounding factors such as urinary sodium [9], and a significant decrease of PTH [6, 7]. Another reason may be the baseline 24-U Ca. As shown in Table 5, significant 24-U Ca levels are found in studies with a lower baseline 24-U Ca. This hypothesis needs further investigation.

The noteworthy finding of our study was similar urinary calcium increase for both repletion protocols, despite more serum 25(OH)D increase in the 50,000 IU group. Both univariate and multivariate analyses showed the same results. In the multivariate analysis, we tried to adjust most of the variables that could affect urinary calcium, including the increase in serum 25(OH)D levels. None of the confounding variables affected 24-U Ca. These results suggest that VDD treatment per se could increase urinary calcium, irrespective of amount of serum 25(OH)D increase. However, this finding should be investigated for treatment protocols that could increase serum 25(OH)D levels more than our study, which is usually in case of patients with severe VDD.

Another noteworthy finding of our study was a similar PTH decrease by both interventions, despite greater serum 25(OH)D increase in the 50,000 IU group. Different studies reveal the inverse correlation between serum 25(OH)D and PTH. Some studies showed that this correlation exists until serum 25(OH)D reaches the level of about 30 ng/mL, and after that, PTH levels begin to plateau [23, 24], however, other studies do not confirm the PTH plateaus at high 25(OH)D concentrations [25].

Studies show that in addition to altering urinary metabolites alone, another way to monitor the treatment of patients with renal stones is to assess the status of 24-h urine supersaturation. Supersaturation is shown to be an acceptable scale for measuring the risk of stone formation [26]. Despite 24-U Ca increase, our findings revealed no significant change in CaOx SS (B = 0.74, p = 0.052) and CaP SS (B = 0.26, p = 0.110). This finding is most likely due to significant increases in 24-h urine volume, magnesium, and citrate as a result of fluid and dietary consultation of patients. In contrary to our result, Vitale et al. [7] showed an increase in CaP SS state. Since 24-h urine volume, magnesium, and citrate are highly affected by dietary intake, controlling the dietary intake of patients could prevent a rise in the supersaturation and stone formation risk.

We evaluated the dietary intakes of our participants to consider their effect on urinary calcium. There was no significant difference in the average intake of micronutrients and macronutrients between the two groups. A noticeable finding was lower than normal calcium and dairy products intake in both groups, although we recommended patients to have a normal calcium intake. Dietary calcium intake could impact serum PTH, 25(OH)D [27] and 24-hour urine calcium. Furthermore, studies to evaluate the impact of different calcium intakes on urinary calcium changes after vitamin D repletion is warranted.

The current study is one of the few clinical trials that evaluated the effects of vitamin D repletion (cholecalciferol) in patients with calcium kidney stones. Another strength of our study was controlling variables that affect urinary calcium and response to vitamin D treatment, such as participants' dietary intake. The main limitation of the current study was lack of a placebo-controlled group which was because of ethical issues. Another limitation was inability of generalizing the results to other conditions, such as severe vitamin D deficiency and severe hypercalciuria, because of study inclusion criteria.

## Conclusions

Although VDD treatment protocols increased 24-U calcium, they did not increase the risk of calcium stone formation assessed by supersaturation state of CaOx and CaP stone formation. Controlling the dietary intake of patients including adequate calcium from dairy products (800–1200 mg/day), salt intake restriction to less than 5 g/day, and consumption of a moderate amounts of animal proteins (0.8–1 g/kg/day) could prevent an increase in supersaturation after 24-U calcium rise due to VDD treatment. Further studies are needed to evaluate the effects of severe VDD treatment on 24-U calcium.

## Data Availability

The dataset supporting the conclusions of this article is available in the http://dregistry.sbmu.ac.ir.

## Abbreviations

25(OH)D: 25-Hydroxyvitamin D
VDD: Vitamin D deficiency
BMI: Body Mass Index
PTH: Parathyroid hormone
24-U: 24-Hour urine
CaOx SS: Calcium oxalate supersaturation
CaP SS: Calcium phosphate supersaturation
